# Profile of HIV-Infected Hispanics with Pancytopenia

**DOI:** 10.3390/ijerph13010038

**Published:** 2015-12-22

**Authors:** Eduardo J. Santiago-Rodríguez, Angel M. Mayor, Diana M. Fernández-Santos, Robert F. Hunter-Mellado

**Affiliations:** Retrovirus Research Center, Internal Medicine Department, School of Medicine, Universidad Central del Caribe, Bayamón, Puerto Rico 00960, USA; amayorb@gmail.com (A.M.M.); diana.fernandez@uccaribe.edu (D.M.F.-S.); robert.hunter@uccaribe.edu (R.F.H.-M.)

**Keywords:** HIV, Hispanics, blood disorders, pancytopenia, antiretroviral treatment, prevalence, mortality

## Abstract

Pancytopenia is seen in late HIV infection; it is associated with medical complications and with decreased survival. We determined the prevalence of pancytopenia at baseline in a cohort of HIV-positive Hispanics living in Puerto Rico, and compared their socio-demographic, immunological and clinical characteristics. A total of 1202 patients enrolled between 2000 and 2010 were included. They were grouped according to pancytopenia status, defined by having: platelets <150,000 μL, white cell count <4000 μL, and hemoglobin <12 g/dL (women) or <13 g/dL (men). Differences were evaluated using Student’s *t*-test, Chi-square test and Kaplan-Meier method. The prevalence of pancytopenia was 8.7%. Patients with pancytopenia had lower BMI and lower CD4 count, as well as higher HIV viral load and higher proportions of unemployment, clinical AIDS and antiretroviral treatment (ART) use (*p* < 0.05). One-year mortality rate was significantly higher in patients with pancytopenia (18.1% *vs*. 5.1%, *p* < 0.001). When stratifying for ART this association persisted for patients who did not receive ART (41.4% *vs*. 5.2%, *p* < 0.001), but it was not seen in patients who received treatment (9.2% *vs*. 5.6%, *p* = 0.196). Pancytopenia was associated with elements of advanced stages of HIV. ART could reduce the mortality of HIV-patients with pancytopenia to levels comparable to patients without the disorders.

## 1. Introduction

Hematological abnormalities are frequently observed in patients with HIV infection. Low blood cell counts, or cytopenias, are the most common of these disorders [1,2]. It has been described that cytopenias, including anemia, leucopenia, and thrombocytopenia, are multifactorial in nature and are associated to an advanced HIV stage, the presence of a high viral load, the use of antiretroviral treatment (ART), the presence of acute and chronic opportunistic infections and finally infiltrative conditions [1,3,4,5]. Cytopenias may present affecting one cell line, or concurrently, involving more than one lineage. The occurrence of universal cytopenia, known as pancytopenia, is usually seen in late stages of HIV infection [5,6]. The presence of isolated cytopenias, and, to a larger degree, pancytopenia, has been associated to a complex spectrum of medical conditions and frequently conveys decreased survival [1,2]. Many studies have described pancytopenia in the context of HIV [2,5,6,7,8,9,10,11]; nevertheless, few studies have evaluated the association of pancytopenia with the sociodemographic profile of subjects, the role of HIV risk practices, the impact of therapeutic interventions, and prognosis.

The purpose of this study was to determine the prevalence of pancytopenia at enrollment in a cohort of HIV-infected Hispanics followed at the Retrovirus Research Center (RRC) in Bayamón, Puerto Rico and to compare their socio-demographic, clinical and immunological characteristics according to the presence or absence of pancytopenia. We also evaluated the association of pancytopenia with one-year mortality and looked for the effect of ART in this cohort of patients.

## 2. Experimental Section

### 2.1. Participants and Setting of the Study

Our study included a total of 1202 HIV-positive adults (21 years and older) who receive their HIV-associated health care at the Ramón Ruiz-Arnau University Hospital or at the HIV ambulatory clinics located in the Universidad Central del Caribe (UCC) in Bayamón, Puerto Rico. RRC sponsors a longitudinal HIV registry where subjects are invited to participate at entry into one of our health care facilities. A detailed explanation of patients’ enrollment and procedures of this cohort study can be accessed elsewhere [12]. Participants of this study had their enrollment between January, 2000 and December, 2010 into the RRC. The UCC’s Institutional Review Board evaluated and approved the study. Written informed consent is obtained from all subjects at entry.

### 2.2. Data Collection Methods and Definition of Pancytopenia and Other Variables Studied

The variables used in this study were obtained from the baseline questionnaire administered to all subjects. The data was obtained via in-person interviews and medical record abstractions. The period covered in the initial questionnaire included data from the preceding 12 months. The laboratory tests used were generally within weeks of the subject entry into the study. During the interviews the following socio-demographic and lifestyles variables were gathered: sex, age, education level completed, employment status, intravenous drug use and alcohol use. The clinical and immunological data, such as: history of AIDS-defining conditions (clinical AIDS), CD4 cell count, HIV viral load, ART use, height, weight and blood cell counts were all abstracted from medical records. ART use was defined as the prescription of at least one antiretroviral medication of any type. Body mass index (BMI) was calculated using the standard formula: [(weight (lb)/height (in)^2^) × (703)]. Patients were classified based on the presence or absence of pancytopenia. The laboratory limits of pancytopenia were defined as follows: hemoglobin (HGB) < 12 g/dL in women and < 13 g/dL in men, white blood cell count (WBC) < 4000 cells/μL, and platelets count (PLT) < 150,000 cells/μL. Mortality information was obtained from the patients’ medical records and revised with data of the mortality registry of the Puerto Rico Health Department, up to December, 2011.

### 2.3. Data Analysis

Descriptive statistics, including the mean, standard deviation (SD), frequencies and proportions, were used to depict the characteristics of the participants of the study and to determine the prevalence of blood disorders. Differences by groups were evaluated using Student’s *t*-test on continuous variables and Chi-square test on categorical variables. The Kaplan-Meier method was employed to assess the association of pancytopenia and one-year mortality. Survival estimates were compared with the log-rank test. Patients who did not have mortality data in the first year after study enrollment were considered as censored. These analyses were also conducted stratifying by ART use. All statistical tests were two-sided and results were considered statistically significant at the 0.05 level. Stata software, version 12.1 (College Station, TX, USA) was used to run the data analysis and to create the figures.

## 3. Results

### 3.1. Description of Participants of the Study and Prevalence of Hematological Disorders

For the entire cohort, the mean (SD) age was 40.3 (10.1) years. The majority of patients were males (67.6%), more than one third (34.9%) reported the use of intravenous drugs and 73.9% identified themselves as unemployed. Forty-one percent of subjects had a CD4 cell count < 200 cells/μL, 8.6% presented clinical AIDS and 62.6% received ART. The prevalence of blood disorders in this cohort at baseline was: 38.2% for anemia, 29.8% for leucopenia and 21.4% for thrombocytopenia. A total of 105 patients (8.7%) presented pancytopenia (Table 1). In general terms, more than half of patients (54.5%) had at least one cytopenia.

### 3.2. Socio-Demographic, Immunological and Clinical Characteristics by Pancytopenia Status

Table 2 shows a comparison of the profile of patients according to the presence or absence of pancytopenia at baseline. Statistically significant differences (*p* < 0.05) were found in the following variables: employment status, BMI, CD4 cell count, HIV viral load, clinical AIDS and ART use. Patients with pancytopenia were more likely to be unemployed (87.5% *vs*. 72.5%), to have lower BMI (mean (SD): 22.0 (4.0) *vs*. 25.1 (5.3)) and have a lower CD4 cell count (0–100 cells/μL: 68.4% *vs*. 23.1%). These pancytopenic patients also had higher HIV viral load (>100,000 copies/mL: 65.0% *vs*. 33.1%), higher clinical AIDS prevalence (24.8% *vs*. 7.0%) and a higher proportion of ART use (72.4% *vs*. 61.6%). Sex, age, level of education, intravenous drug use and alcohol use were not statistically different according to the presence of pancytopenia (*p* > 0.05). In Table 3 appear the results of the prior analysis but stratified by ART use. We demonstrate that irrespective of ART use, patients with pancytopenia, have a lower CD4 cell count, lower BMI, higher HIV viral load and higher clinical AIDS prevalence (*p* < 0.05).

**Table 1:** Description of study participants and prevalence of cytopenias (n = 1202).

Variables	%
Males	67.6
Age, mean (SD)	40.3 (10.1)
Intravenous drugs use	34.9
Unemployment	73.9
CD4 cell count <200 cells/μL	41.0
Clinical AIDS	8.6
ART use	62.6
Anemia	38.2
Leucopenia	29.8
Thrombocytopenia	21.4
Pancytopenia	8.7

ART = Antiretroviral treatment.

**Table 2:** Comparison of socio-demographic, clinical and immunological characteristics of study participants by status of pancytopenia.

Variables	Pancytopenia (n = 105)	No Pancytopenia (n = 1097)	*p*-Value
*Males*, *%*	67.6	67.6	0.997
*Age*, *mean (SD)*	41.7 (8.9)	40.1 (10.2)	0.088
*Education*, *%*			
< HS	35.6	30.9	0.606
HS	37.5	39.4	
College	26.9	29.7	
*Unemployment*, *%*	87.5	72.5	0.001
*Intravenous drugs use*, *%*	37.1	34.6	0.607
*Alcohol use*, *%*	56.7	50.0	0.187
*BMI*, *mean (SD)*	22.0 (4.0)	25.1 (5.3)	< 0.001
*CD4 cell count*, *mean (SD)*	111.7 (140.7)	345.1 (293.0)	< 0.001
*CD4 cell count (cells/μL)*, *%*			
0–100	68.4	23.1	< 0.001
101–199	14.3	14.2	
≥200	17.3	62.7	
*HIV viral load (copies/mL)*, *%*			
<10,000	10.0	35.3	< 0.001
10,000–100,000	25.0	31.6	
>100,000	65.0	33.1	
*Clinical AIDS*, *%*	24.8	7.0	< 0.001
*ART use*, *%*	72.4	61.6	0.030

HS = High school; BMI = Body mass index; ART = Antiretroviral treatment.

**Table 3:** Comparison of selected clinical and immunological characteristics of study participants by ART use and status of pancytopenia.

Variables	ART^+^			ART^−^		
	Pancytopenia (n = 76)	No pancytopenia (n = 676)	*p*-Value	Pancytopenia (n = 29)	No Pancytopenia (n = 421)	*p*-Value
*BMI*, *mean (SD)*	22.5 (3.9)	24.8 (4.9)	< 0.001	20.7 (4.7)	25.8 (5.8)	< 0.001
*CD4 cell count*, *mean (SD)*	95.6 (100.4)	256.7 (245.5)	< 0.001	167.6 (226.3)	491.6 (306.5)	< 0.001
*CD4 cell count(cells/μL)*, *%*						
0–100	71.0	31.2	< 0.001	59.1	9.6	< 0.001
101–199	14.5	18.2		13.6	7.7	
≥200	14.5	50.6		27.3	82.7	
*HIV viral load (copies/mL)*, *%*						
<10,000	9.3	30.2	< 0.001	12.0	43.6	< 0.001
10,000–100,000	24.0	28.9		28.0	36.2	
>100,000	66.7	40.9		60.0	20.2	
*Clinical AIDS*, *%*	17.1	8.3	0.012	44.8	5.0	< 0.001

ART = Antiretroviral treatment; BMI = Body mass index.

### 3.3. Relationship between One-Year Mortality, Pancytopenia and ART

**Figure 1:**
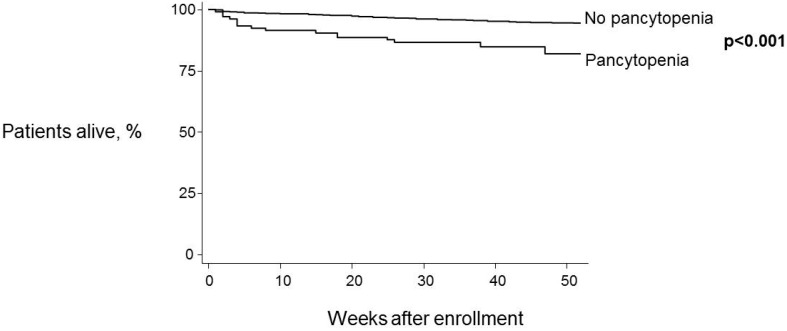
One-year survival estimates by pancytopenia status at baseline. Log-rank test was used to compare curves.

**Figure 2:**
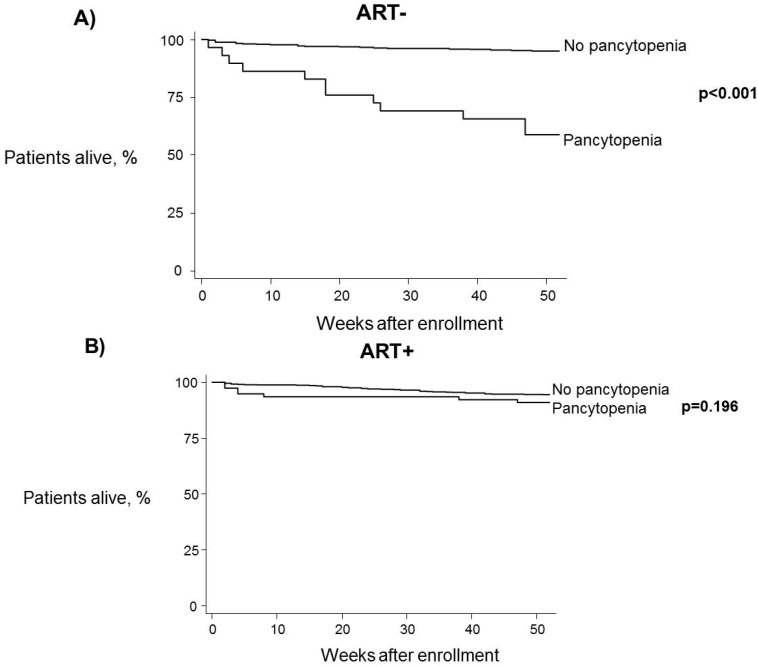
One-year survival estimates by pancytopenia status at baseline, controlling for antiretroviral treatment use. (**A**) Patients not receiving ART; (**B**) Patients receiving ART. Log-rank test was used to compare curves.

One-year mortality rate was significantly higher in patients with pancytopenia at baseline (18.1% *vs*. 5.1%, log-rank test: *p* < 0.001) (Figure 1). When stratifying for ART this association persisted for patients who were not on ART (41.4% *vs*. 5.2%, log-rank test: *p* < 0.001) (Figure 2A), but was not seen in patients receiving antiretroviral therapy (9.2% *vs*. 5.6%, log-rank test: *p* = 0.196) (Figure 2B).

## 4. Discussion

The presence of hematological abnormalities in particular cytopenias associated to HIV was documented in the early years of the epidemic [7,8,13]. Advances in the understanding of the infection and its relation to hematologic homeostasis have improved over the last 10 years. The majority of studies on this topic have focused on anemia, the most common of these disorders [1,4,6,14]. When compared with anemia, leucopenia and thrombocytopenia have been described at a lesser extent, and the concurrent observation of the three, pancytopenia, is the least investigated (a PubMed search conducted on August 10, 2015 (including the “blood abnormality” and HIV) retrieved 2892 results for anemia, 1202 for thrombocytopenia, 928 for leucopenia and 188 for pancytopenia). Our study was directed to understand, not only the prevalence of pancytopenia in a group of HIV-infected patients, but to contribute by defining the traits which may be associated to those patients with low blood cell counts. We have assessed how pancytopenia relates to other clinical, socio-demographic and immunological variables and with patients’ one year survival.

The prevalence of pancytopenia for the HIV-positive patients of this study (8.7%) was higher than the reported in other investigations, which ranged between 0.3% and 6.0% [2,6,10,11]. Differences on these values can be attributed to two main reasons: the blood cells parameters used to define pancytopenia and the stage of the HIV infection of patients (*i.e*., their immunological and ART status) at the time the studies were conducted. Of the four epidemiological studies cited evaluating the prevalence of pancytopenia in their cohorts, only one had the definition that we used to determine pancytopenia [10]. Two other studies [2,11] had more strict cutpoints and used lower values in their definitions; for example: HGB < 10 g/dL, WBC < 2750 cells/μL and PLT < 125 cells/μL. In addition, there were discrepancies in the design of the studies, specifically in the type of HIV patients that were included. In the studies conducted in Uganda [2] and Ethiopia [6], patients were initiating ART therapy: some were HAART-naïve and others had used HAART for ≤6 months (% pancytopenia: 0.5 and 0.7, respectively). The study carried out in India [10] had 33% of patients receiving ART as well (% pancytopenia: 6.0). In contrast, a multisite study of HIV-infected patients from Asia, Africa and the Americas, only measured pre-ART hematological abnormalities (% pancytopenia: 0.3) [11]. If ART is associated with pancytopenia, as our study and others [1,4] have pointed out, including subjects on ART therapy will increase the prevalence of blood disorders particularly cytopenias.

Multiple studies have suggested that cytopenias tend to occur in patients with advanced stage of HIV infection. At this point in the natural history of the disease, the immune system is substantially debilitated, viral replication has not been controlled and opportunistic infections are rampant [1,2,4,5,15]. In our study, patients with pancytopenia were more likely to have all these elements of advanced HIV infection, as compared to those without pancytopenia. An important event seen in our group of subjects with pancytopenia was ART use, as a significantly higher proportion of these patients were on treatment. This finding might concur with literature that indicates ART can induce cytopenias [4,15]. However, because of the design of this study, in which only baseline data was analyzed, we cannot ascertain that pancytopenia was a result of ART. Pancytopenia might also be related to other factors of the HIV infection, or other co-morbidities, such as Hepatitis C or cirrhosis of the liver. On the other hand, the opposite may also be true, that ART was instituted due to the advanced nature of the infection including the co-existence of the cytopenias. The finding that 29 patients with pancytopenia were ART-naïve establishes that in at least 27.6% of subjects the cytopenias were not related to ART therapy. Finally, we can also affirm that in the immunological and clinical variables commonly used as indicators of late HIV, patients with pancytopenia on ART had the same outcomes that those without ART (Table 3).

Isolated cytopenias have been associated with increased risk of death in HIV-infected individuals [12,16,17,18]. In our study we have shown that patients with pancytopenia had a higher one-year mortality rate than those without pancytopenia. When we controlled for ART use, this association persisted only for patients who were ART-naïve at baseline. Studies suggest that ART therapy may improve cytopenias in selected group of patients [1,6,15]. Longitudinal analysis of our cohort may provide further data in this particular issue. It is quite possible that the one-year survival observed in subjects with pancytopenia and ART is related to the therapeutic improvement of the cytopenia associated to ART. We plan to perform these studies in the near future.

## 5. Conclusions

Patients with pancytopenia were more likely to have factors related to an advanced stage of HIV infection, but only those not receiving ART showed a poorer outcome after one year. The use of ART in patients with pancytopenia could reduce their mortality to levels comparable to patients without the blood dyscrasia. Future studies on this cohort should evaluate whether the administration of ART is associated with correcting cytopenias. Additionally, a prospective study should evaluate the mortality risk of patients with blood disorders after controlling for other factors associated with mortality during HIV. Efforts to establish specific etiologies are also required to prescribe these patients the appropriate treatment in order to improve the hematologic status, and consequently their survival.
